# Physical Agent-Based Treatments for Overactive Bladder: A Review

**DOI:** 10.3390/jcm11175150

**Published:** 2022-08-31

**Authors:** Nurida Khasanah, Hung-Yen Chin, Chih-Wei Peng

**Affiliations:** 1 Graduate Institute of Biomedical Materials and Tissue Engineering, College of Biomedical Engineering, Taipei Medical University, Taipei 11031, Taiwan; 2 Department of Obstetrics and Gynecology, Faculty of Medicine Public Health and Nursing, Universitas Gadjah Mada-Dr Sardjito General Hospital, Yogyakarta 55281, Indonesia; 3 Department of Obstetrics and Gynecology, Taipei Medical University Hospital, Taipei 11031, Taiwan; 4 Department of Obstetrics and Gynecology, School of Medicine, College of Medicine, Taipei Medical University, Taipei 11031, Taiwan; 5 School of Biomedical Engineering, College of Biomedical Engineering, Taipei Medical University, Taipei 11031, Taiwan; 6 International Ph.D. Program in Biomedical Engineering, College of Biomedical Engineering, Taipei Medical University, Taipei 11031, Taiwan; 7 School of Gerontology and Long-Term Care, College of Nursing, Taipei Medical University, Taipei 11031, Taiwan; 8 Research Center of Biomedical Device, Taipei Medical University, Taipei 11031, Taiwan

**Keywords:** physical-based agent, overactive bladder, neuromodulation, electrical stimulation, magnetic stimulation, laser, low-intensity shock energy

## Abstract

Almost one-fifth of the people in the world experience a decrease in quality of life due to overactive bladder (OAB) syndrome. The main bothersome symptoms are urgency accompanied by urinary frequency and nocturia. This chronic, disabling condition is first managed by reducing fluid intake and pelvic floor muscle training, supplemented with antimuscarinic drugs, if necessary. However, refractory cases often still occur. In more severe cases, invasive surgical interventions can be considered; yet, the success rate is still inconsistent, and there is a high complication rate. This condition is frustrating for patients and challenging for the medical staff involved. Although its pathophysiology has not been fully elucidated, peripheral autonomic somatic and sensory afferent receptors are considered to be involved in this condition. Hence, currently, physical agent-based treatments such as neuromodulation have taken a significant place in the third-line therapy of OAB. The efficacy and safety profiles of electrical and magnetic stimulation continue to evolve. Physical-based agents provide an appealing option owing to their effectiveness and minimal side effects. In addition, more physical therapies using light and shock energy are currently being investigated. Thus, a comprehensive understanding of these modalities is an extremely important aspect to provide the most suitable modalities for patients.

## 1. Introduction

Overactive bladder (OAB) is a type of urinary incontinence syndrome experienced by many more women than men [1]. Although the risk increases with age, especially after 40 years [2], it can affect both children and young individuals as well. Based on the International Consultation on Incontinence Research Society (ICI-RS), a diagnosis of OAB is made if someone experiences voiding urgency, frequency, and nocturia that may or may not be accompanied by urinary incontinence, with no proven infection or other obvious pathology [3].

The prevalence rate of OAB has been reported to be 20.8% in Asia and up to 11.8% of the population in Western countries [4,5]. OAB is not lethal; however, it significantly affects patients’ quality of life in many aspects [1]. Patients with OAB spend approximately US 267 every year on their treatment [6]. Some research also reported lower work productivity and higher levels of anxiety and depression in OAB patients. In addition, their sexual well-being may also be affected [7].

OAB is highly difficult to treat due to the complex nature of OAB’s pathophysiology. The suggested first-line therapy is behavioral therapy that includes reducing fluid intake by 25%, avoiding caffeine and diuretics, and training the pelvic floor muscles [8]. Antimuscarinic medication is suggested as a second-line treatment; however, relapse is seen in up to 60% of patients after 3 months of drug cessation [9]. Furthermore, anticholinergic medications are discontinued in many cases due to unpleasant dry mouth and safety problems for older patients such as cognitive impairment and cardiac consequences [10]. A botulinum toxin injection is another option for OAB with suboptimal persistence; yet, it increases the risk of urinary tract infections and urine retention [11].

**Figure 1:**
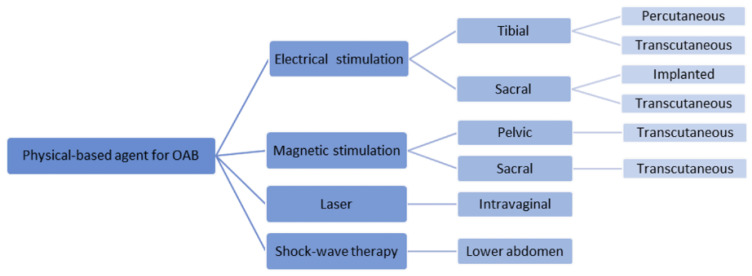
Classification of physical-based agents as third-line options for overactive bladder (OAB). The classification is followed by the stimulation area and the way it is applied.

As a third-line therapy, there are at least four classifications of modalities, as shown in Figure 1, namely, electrical stimulation, magnetic stimulation, laser therapy, and shock wave therapy. Electrical stimulation can be applied to either the sacral or tibial area, while magnetic stimulation is recommended to be placed in the pelvic or sacral area during treatment. Transcutaneous or percutaneous approaches are available options for electrical stimulation, and the efficacy and safety of each method continue to be investigated. It is important to explore promising options which are expected to be effective, non-invasive, and to have minimal side effects. Hence, physicians should understand all of the many available third-line modalities in order to correctly choose them for patients. In this review, we provide comprehensive information on physical agent-based third-line treatments for OAB as well as updated information on each physical agent and possible future modalities.

## 2. Electrical Stimulation Modalities

Electrical stimulation uses low-frequency electricity to elicit action potentials that travel along nerves and then activate afferent fibers which may influence important sites of the central nervous system (CNS) [12].

Efferent nerve-fibers then transport the stimulus to the necessary pelvic organs. Thus, this treatment is known as neuromodulation, as it does not involve the direct stimulation of the organ but instead has an indirect effect on the entire micturition system [13]. Below, we introduce several of the most often utilized electrical stimulation modalities and provide detailed information about them.

### 2.1. Percutaneous Sacral Nerve Stimulation (P-SNS)

The sacral neuromodulatory technique involves posteriorly modulating the sacral nerve root (S3) through the neuro-foramen. Tanagho and Schmidt, in the late 1980s, were the first to develop this technique by introducing the idea of a “bladder pacemaker” [14]. The postulated mechanism of action is that P-SNS alters the somatosensory pathway from the sacral to the cortical area [15]. The P-SNS device, named InterStim^®^, received US Food and Drug Administration (FDA) approval in 1999 for treating urinary urgency-frequency and non-obstructive urinary retention [16]. The implantation of P-SNS needs two-stage surgery. If the number of incontinence episodes is reduced by at least 50% by using the temporary electrode in stage 1, then the second stage of device implantation would be carried out [17].

P-SNS is superior to oral medical therapy for treating OAB [18]. The InSite study randomized 147 OAB patients who had failed conservative or pharmacological treatments and allocated them to either a P-SNS or pharmacological treatment (PT) group. The intention-to-treat model found that the OAB therapeutic success rate at 6 months is 61% for P-SNS compared to 42% for PT (*p* = 0.02) [18]. P-SNS significantly reduces the frequency and number of urgency episodes and the number of urinary incontinence episodes [19]. Moreover, this device appears to be effective for the long term, with sustained quality of life improvements and an acceptable safety profile through 3–5 years in OAB subjects [20,21].

Considering that the device requires minor surgery for implantation, there are several complications including dislocated leads, device malfunction, and dysesthesia [21]. The required revision surgery and battery replacement vary in up to 39% of cases [22].

The true mechanism of action of P-SNS is not fully elucidated, but in the last two decades, a lot of studies were conducted to investigate its mechanism of action to activate or inhibit neural reflexes associated with lower urinary tract function [23,24]. It seems that P-SNS modulates spinal reflexes and brain activity. Using a neurophysiological evaluation, Malaguti, et al. [15] found that alterations in pudendal somatosensory evoked potentials in 15 of 24 patients who underwent P-SNS. It was revealed that P-SNS alters the somatosensory pathway from the sacral to the cortical area. Moreover, Gill, et al. [25], through a magnetic resonance imaging (MRI) brain examination, confirmed that sacral neuromodulation influences brain activity in women with OAB. This response varies with different durations of use. Another study reported that P-SNS inhibits the detrusor muscle without influencing urethral resistance or detrusor contractility during the voiding phase [26].

Recently, InterStim^®^ has undergone significant refinements and improvements, including a smaller implantable pulse generator (IPG) and the use of wireless test programmers [27]. Furthermore, a novel, remotely programmed P-SNS system named BetterStim (PINS, Beijing, China) was manufactured to reduce frequent postoperative follow-up. Through a provided web browser, physicians can obtain detailed information about their patients and stimulators as well as remotely control the stimulator. BetterStim would be especially beneficial for patients who live far away from a hospital [28].

### 2.2. Transcutaneous Sacral Nerve Stimulation (T-SNS)

T-SNS consists of transcutaneous electrical nerve stimulation (TENS) placed over the parasacral region at the level of the S3 foramen [29]. This technology has been extensively examined in children, with positive results for treating OAB; however, only a few studies have been undertaken in adult patients. Stimulation is conducted on two or three alternate days per week for 4~12 weeks [30,31,32]. Veiga, et al. [33] successfully assessed that this method could also be effective after twice-weekly sessions if the patients could not attend three times weekly.

Unmyelinated C-fibers are postulated to mediate bladder overactivity. Hence, transcutaneous electrostimulation non-invasively activates myelinated alpha afferent fibers (large-diameter nerves) to inhibit second-order neurons in the dorsal horn of the medulla, which in turn prevents impulses carried by C-fibers, thereby decreasing detrusor muscle activity [34]. Furthermore, another mechanism proposed that transcutaneous electrostimulation stimulates endogenous opioid release, which activates inhibitory descending systems to suppress bladder contraction [35].

In children, T-SNS has obvious positive short- and long-term follow-up effects. Incontinence symptoms were completely resolved in 77% of patients after 20 sessions of T-SNS [31]. The newest randomized study in children revealed that T-SNS is more effective than anticholinergic with a complete clinical resolution rate in a larger number of patients [36]. In contrast, another study found the combination of T-SNS and anticholinergic is better than monotherapy in children with urge incontinence [37]. Long-term complete responses were seen in 73% of patients with 2 years of follow-up after a previous maximum of 20 T-SNS sessions [31].

Padilha, et al. [30] and Jacomo, et al. [38] compared the effect of T-SNS and transcutaneous tibial nerve stimulation (T-TNS) in treating OAB, and they revealed different results. The former found that both procedures were equally effective in improving OAB symptoms, while the latter found that T-SNS only reduced the nocturia episodes. The difference might have been due to the electrostimulation parameters used in the studies and the participants’ characteristics. Padilha, et al. [30] enrolled adult women, while elderly women with a mean age of 68 years were subjects in the research of Jacomo, et al. [38]. No side effects were noted in children, adolescents, adults, or the elderly after T-SNS application [30,38].

### 2.3. Percutaneous Posterior Tibial Nerve Stimulation (P-TNS)

**Figure 2:**
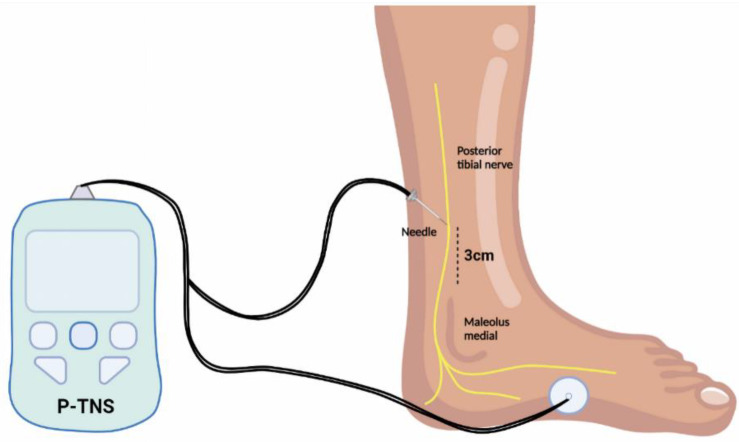
Illustration of the electrodes and needle position for percutaneous posterior tibial nerve stimulation (P-TNS) therapy. The needle is inserted parallel to the tibial nerve.

P-TNS was described after SNS was demonstrated to develop less-invasive techniques of neuromodulation. The technology was inspired by acupuncture techniques that stimulate the posterior tibial nerves used in traditional Chinese medicine practices [39,40]. As for the term itself, P-TNS is a type of peripheral electrical stimulation that modulates the S3 nerve plexus, which controls bladder function, through the tibial nerve in a retrograde manner. The tibial nerve originates from L4-S3 nerve roots, which are the same spinal segments as those in the bladder innervation [41]. P-TNS is less invasive than the P-SNS technique. Once the current is applied through the needle electrode (Figure 2), the flexion of the big toe or the movement of the other toes confirms the correct positioning of the needle electrode [42].

In 2000, the US FDA approved the use of P-TNS as an office therapy for treating OAB. According to the European Association of Urology guidelines for urinary incontinence in adults, women for whom antimuscarinic medication has failed should benefit from P-TNS [43].

P-TNS increases the bladder capacity and delays the onset of detrusor overactivity [44]. The efficacy of P-TNS in improving OAB symptoms was successfully demonstrated in several studies. In 2010, Peters et al.—in the SUmiT Trial, with a total of 220 participants—described that P-TNS significantly improved bladder symptoms (54.5%) compared to the sham group (20.9%). In addition, the success rate of P-TNS in reducing frequency, night-time voiding, and urgency incontinence was about 60~70% [45]. A recent study found that the quality of life of patients treated with P-TNS is better than that for those treated with the antimuscarinic drug (Solifenacin); however, the combination of both results in significant improvements that are superior to those of one modality [46]. There is no difference in effectiveness when the number of days per week of P-TNS administration is increased [47].

The interesting advantage of P-TNS is the long-term effect, in which patients still experience OAB improvement even when the nerve is not being stimulated. It was proven by a STEP (Sustained Therapeutic Effects of Percutaneous Tibial Nerve Stimulation) study that, after 36 months of therapy with an average of 1.1 therapy sessions per month, 77% of patients sustained OAB improvements [48].

No serious adverse effects have been reported [48]. However, in a real setting, Du et al. [49] reported that, after 1 year of therapy, only 39.5% of patients were willing to maintain P-TNS therapy owing to various reasons, such as the need for repeated visits. Hence, a new implantable P-TNS device such as the Bluewind RENOVA system, Bioness Stimrouter, and eCoin was introduced so that patients can repeat the treatment by themselves at home. Several studies reported good results of this being safe and well-tolerated for up to 9 years [40].

### 2.4. Transcutaneous Tibial Nerve Stimulation (T-TNS)

Over the past decade, posterior TNS has become a well-accepted and relatively effective third-line therapeutic option for patients with OAB. The development of T-TNS began in 1983 when McGuire et al. were the first to effectively demonstrate the inhibition of detrusor contractility via a positive transcutaneous electrode to the common peroneal nerve in patients with recurrent OAB [50]. T-TNS has similar clinical efficacy to that of P-TNS; yet, it is more comfortable and has a shorter preparation time compared to P-TNS [43]. No local or systemic adverse effects related to T-TNS have been reported [51].

Through adhesive surface electrodes, similar to P-TNS, this device modulates the innervation system of the lower urinary tract in a retrograde manner. Current is used to inhibit contractions of the detrusor muscle to reduce urination frequency [52].

T-TNS can be performed on one or both legs for 30 min The initial session takes place in a hospital, but subsequent regimens can be completed at home [53].

Several clinical trials applying T-TNS showed that 50~95% of patients experienced OAB symptom reduction [43,51]. After 6 weeks of treatment, Sonmez et al. [43] reported a reduction in the severity of incontinence (80.7 to 5.46), the frequency of voiding (11.5 to 6.8), incontinence episodes (4.4 to 0.7), and nocturia (3.2 to 1.3), and OAB-V8 symptom severity (27.5 to 4.4). In contrast, ELECTRIC trials found that T-TNS is not effective for people with poor cognitive capacity and limited independent mobility. However, there are some missed primary outcome data due to limitations in 24 h pad collections [54]. The frequency of stimulation (once or twice a week) had a no significant effect on the effectiveness of lowering OAB symptoms [55]. Moreover, Alkis et al. [53] found that a three-times-a-week treatment resulted in a more-rapid decrease in OAB symptoms compared to a once-a-week treatment.

Another thing to pay attention is whether stimulation is better given in one or both legs. In one study, the stimulation of one leg was adequate to relieve OAB symptoms, except for nocturia, but the best results were seen with the two-leg protocol [56]. Therefore, the use of T-TNS should be tailored to the specificity of the symptoms described by the patient.

T-TNS was found to have a long-term satisfactory effect at 12 months of follow-up in 80.5% of patients [51]. In recent years, some companies have developed novel ambulatory T-TNS devices. T-TNS can be performed by the patient or their family at home after receiving suitable training [53]. Patients do not have to go to a hospital as often, which is expected to encourage them in continuing a longer-term treatment. Moreover, since it is completely non-invasive, the device is proven to be effective and also well tolerated by children [57].

## 3. Magnetic Stimulation Modalities

An electrical current produced by a magnetic field was discovered more than a hundred years ago by Michael Faraday. Since then, researchers have tried to use magnetism to treat illnesses. Its initial application to humans was developed at the University of Sheffield, in the United Kingdom (UK), with a demonstration of human cortical stimulation in 1985 [58]. High-frequency stimulation (of >5 Hz) generates excitatory effects in the brain, whereas low-frequency stimulation (below 1 Hz) produces inhibitory effects [59]. The influence of peripheral magnetic stimulation remains inconclusive.

Magnetic and electrical stimulation are assumed to work in similar ways. Magnetic stimulation, however, does not require the direct induction of an electric current into the body. It is a completely non-invasive technique that delivers a rapid-pulse, high-intensity magnetic field to the body. Eventually, it causes a voltage differential between two sites and produces an electric field, causing electrons to flow between the two locations [60]. The magnetic field can pass through any medium, including vacuum space, with no energy loss. As a result, deep-tissue penetration is possible. Because of this feature, no mechanical contact is required, making it suitable for people with severe skin hypersensitivity or allodynia. The patient does not need to undress because the magnetic field can flow through clothing. Furthermore, magnetic stimulation rarely causes pain owing to no charged particles being injected into the skin and superficial tissues [58,60]. Following that, we present two distinct types of magnetic stimulation methods and also provide thorough information on them.

### 3.1. Pelvic Magnetic Stimulation (P-MS)

P-MS is a sort of magnetic stimulation in which the coil is installed on an armchair-style seat, and the participant is encouraged to sit in such a way that the anus is positioned in the center of the coil and the anal sphincter contracts the most during stimulation [61]. The patient does not need to undress and appears to experience little cutaneous discomfort. Because of that, this technique is thought to be suitable for children compared to electrical stimulation. [62].

P-MS is hypothesized to reduce OAB by relaxing detrusor smooth muscles via the activation of pudendal nerve afferents, thereby “blocking” the parasympathetic route at the spinal cord, inhibiting hypogastric sympathetic neurons, or a combination of both methods [63,64]. Other literature also mentioned that a rapidly pulsed magnetic field on target tissues induces tiny eddy currents to flow into tissues, causing the depolarization of nerve axons. The depolarization of corresponding muscle fibers causes those fibers to contract. The motor end-plates tend to gain muscular strength and endurance if this occurs on a regular basis [63,65].

According to research, the best frequency for stimulating the detrusor smooth muscle is 10 Hz, whereas 20~50 Hz stimulates striated muscles in the pelvic floor and promotes urethral closure [64,65]. Each treatment was separated by a rest period of 36~72 h to prevent muscle fatigue [64].

After 16~18 sessions of P-MS treatment, the cure rate of OAB was 56.3~61.7% [65,66]. Active treatment significantly decreased voiding episodes in 24 h by 42.8% and the number per week by 44.2% [66,67]. More than one-third of patients could maintain the therapeutic effect on urgency, frequency, and urgency incontinence for up to 24 weeks [66]. OAB symptoms were even reported to have disappeared in 15.8% of female patients and in 5.3% of male patients [67]. In addition to a qualitative assessment using a validated questionnaire, Vadalà, et al. [68] reported urodynamic changes after P-MS treatment. It exhibited significant (*p* < 0.01) increases in the cystometric capacity (147% ± 51.3%), maximum urethral closure pressure (MUCP) (110% ± 34%), and urethral functional length (99.8% ± 51.8%) values compared to the baseline [68].

Beyond the benefits of P-MS, the sole cause for noncompliance with this therapy is the need to visit the clinic on a frequent basis, which may be due to a geographical element or a lack of financial resources [65]. Although studies proved the safety, it is important to note that P-MS is contraindicated for persons who have tattoos or a pacemaker or are pregnant [69].

Until now, there have been fewer publications regarding the use of magnetic stimulation over the pelvic area for OAB than for stress urinary incontinence (SUI). Most of the studies applied this machine for SUI since it causes contractions of the pelvic floor similar to stimulating Kegel exercises and increases urethral pressure to reduce SUI symptoms. In Japan, Kobayashi et al. [69] reported that P-MS is covered by health insurance for female patients who suffer from intractable OAB and do not improve after taking medication.

### 3.2. Sacral Magnetic Stimulation (S-MS)

The use of a magnetic coil is not limited to the pelvic area. The coil can be situated unilaterally or bilaterally over the sacral S2–S4 area and is so-called S-MS. However, only a few studies have discussed the impacts of S-MS on urinary incontinence.

S-MS is designed to modulate the S2–S4 nerve roots, which is thought to be an efficient way to modulate both the pelvic floor and pelvic organs [70]. The right location of the coil was confirmed by the contraction of intrinsic muscles of the foot and the urethral and anal sphincters [70,71,72].

S-MS exhibited a significant increase in the mean bladder capacity in 48 patients who underwent repetitive S-MS of 15 Hz at a 50% maximum output intensity for 30 min. The urine volume per void and quality of life score improved by 3- and 3.5-fold, respectively, compared to the sham stimulation group. The mean number of leaks significantly decreased from 5.3 ± 6.7 to 1.6 ± 3.2 in the group (*p* = 0.01) with active treatment for 3 days [71]. One study demonstrated a better outcome of 20 sessions of S-MS compared to 20 sessions of transcutaneous sacral electrical nerve stimulation for neurogenic OAB [72]. However, no study has examined the long-term effects of S-MS on OAB conditions. There is still a lack of high-quality data supporting the efficacy, safety, and long-term benefits of S-MS for OAB.

The coil for S-MS is bulky, which might not be interesting for many people to study since there is a magnetic chair. However, considering that magnetic stimulator machines are expensive, we inferred that having only one magnetic stimulator machine might be useful for several medical indications at once—for example, peripheral magnetic stimulation for incontinence and transcranial magnetic stimulation for depression and other medical conditions. Hence, this could be a good opportunity to do research in the future.

## 4. Potential New Therapeutic Technologies

### 4.1. Laser Therapy for OAB

The principle of laser therapy to treat OAB differs from both electrical stimulation and magnetic stimulation. Laser therapy uses intensely focused specific wavelengths of light to change or stimulate the structure to which it is exposed—in OAB cases, the lower urinary tract mucosa [73].

In most postmenopausal women, OAB results from the gradual decrease in estrogen levels, resulting in vulvovaginal atrophy (VVA) and the loss of urethral and bladder elasticity. Due to the limited number of clinical trials, the mechanism of the laser for improving OAB is still questionable. A vaginal laser is expected to reactivate the extracellular matrix and collagen formation in the deeper layers of the vaginal wall, which might extend to the urethra and bladder, thus resulting in a considerable reduction in urogenital aging symptoms [74].

Perino et al. [74], in their previous study, were the first to discover unexpected benefits when using a CO_2_ laser to treat VVA, with some patients reporting a concurrent significant reduction in urine urgency or frequency. Then, they started to evaluate the possible role of fractional CO_2_ laser vaginal treatment in reducing OAB symptoms in 30 postmenopausal women. The laser pulses are delivered over the vaginal wall using a specific intravaginal probe that protects the uterine cervix from being exposed. The CO_2_ laser is distributed, focusing on small spaces (called DOT spacing) to cover all of the vaginal area. All patients underwent only three sessions of treatment, separated over a period of at least 30 days [74]. Their results indicated that the number of micturition and urge episodes was significantly reduced (*p* < 0.0001). There was a substantial reduction in incontinence events in nine individuals with wet OAB (*p* < 0.0001). However, there was no randomization or control group in that study [74].

Subsequently, Aguiar compared the use of a CO_2_ laser, vaginal estrogen, and vaginal lubricant to treat OAB in 72 postmenopausal women. This was a randomized study that showed a significant reduction in the International Consultation on Incontinence Questionnaire Overactive Bladder (ICIQ-OAB) scores after applying a CO_2_ laser, even compared to vaginal estrogen, which is known as the standard therapy for VVA. There was no adverse event during the study [75].

Moreover, several researchers explored the potential use of a less-ablative type of vaginal laser to treat OAB. The erbium: YAG laser considerably improved OAB symptom scores (OABSSs) [76,77]. However, the symptom reduction was not sustained at 12 months of follow-up [76]. In Japan, Okui [78] compared the erbium: YAG laser to sling surgery to treat mixed urinary incontinence. Exacerbation was exhibited in surgical groups, while a significant improvement was seen in the laser therapy group. They also compared laser therapy to anticholinergics and β3-adrenoceptor agonist medication and found comparable improvements in OABSSs between the laser group and women who received standard OAB medication [77].

In clinical trials, laser treatment sessions were conducted in two or three sessions for 3 months, which was less than the duration of electrical or magnetic stimulation. Furthermore, no side effects from fractional CO_2_ laser or erbium: YAG laser therapy were seen [74,76,77]. While vaginal laser therapy appeared to have a positive effect, compared to previous well-known modalities, the evidence for its use is so far inadequate owing to single-center small population research with no long-term assessments. The use of this method is now limited to clinical trials.

### 4.2. Low-Intensity Extracorporeal Shock Wave (Li-ESW)

Li-ESW transfers lower-intensity (0.2 mJ/mm^2^) energy than typical ESW lithotripsy (ESWL), which usually is used to disintegrate urinary tract stones. The tissue between the shockwave generator and the target suffers minimal damage [79]. Interestingly, instead of causing damage to the target, they discovered that Li-ESW promotes angiogenesis and tissue regeneration and decreases inflammation [79]. The clinical application of Li-ESW for various incontinence disorders including OAB has been recently explored.

Lee et al. [80] published a preliminary study in 2019 that demonstrated considerably improved functional bladder capacity and daytime frequency and decreased post-voided residual urine and urgency in 82 females with OAB. Furthermore, Lu et al. [81] used the same parameter in 65 patients and observed the same outcomes compared to a sham group, with no adverse side effects. As the pathophysiology of OAB is also correlated with estrogen deficiency, Lin et al. [82] studied rats with OAB induced by ovarian hormone deficiency. They proposed the potential mechanism of Li-ESW to enhance OAB by enhancing tissue regeneration and bladder repair through anti-inflammation, urothelium proliferation, increased angiogenesis, and smooth muscle remodeling in the bladder. The study continued to test Li-ESW in 58 postmenopausal women who have LUTS-related urogenital atrophy and found that Li-ESW reduced daytime frequency, nocturia, and urgency. Clinical investigations of OAB used a probe that was inclined 45° and applied over the suprapubic area with an intensity of 0.25 mJ/mm^2^, 3000 pulses, and 3 pulses/second once weekly for 8 weeks. Validated OAB symptoms and life bothersome questionnaires, a 3-day urinary diary, and uroflowmetry were utilized in the research [80,81].

Compared to other well-known modalities, although there is a prospective randomized clinical trial for the use of Li-ESW in OAB, the study still lacks high quality data describing the safety, efficacy, and long-term benefits for OAB patients.

## 5. Challenges Ahead

Physical agents provide a greater effectiveness while avoiding the negative effects of drugs and botulinum toxin injections. Clinical trials demonstrated the superiority of neuromodulation using P-SNS compared to oral medication therapy (61% vs. 42%, *p* = 0.02) [18]. In addition, at 10 years, P-SNS provides an improvement in the quality of life and is cost-effective compared to botulinum toxin injections or continued oral medication [83].

Despite the fact that all physical therapies are safe and efficacious compared to other treatment options, larger clinical trials are needed to assess the head-to-head performance of each physical agent. The number of treatment sessions to reduce symptoms is important to be considered. There are still certain flaws to consider, such as the fact that P-TNS and S-NS are both quite invasive procedures that need precise needle penetration into an exact place, and most of the technology requires patients to regularly attend a clinical setting.

Table 1 should help clinicians decide on the best option for their patients. Choosing a specific device according to the complaints experienced by the patient is the most important thing that must be considered. Moreover, healthcare professionals and patients must consider the costs, treatment effectiveness, unpleasant effects, and hospital attendance, since these are all factors that contribute to the rate of medical treatment persistence. As a result, new user-friendly technologies are needed to satisfy these demands. Current research is focusing on developing smaller, rechargeable implanted devices that patients can use at home under the supervision and guidance of a urologist or gynecologist instead of in a doctor’s office.

## 6. Conclusions

There are now various promising options as third-line treatments for OAB. The technology should be chosen depending on the symptoms, the cost-effectiveness, the onset effect, and the long-term impacts. If patients are satisfied with the device, compliance will increase. Breakthroughs are projected to become preferred-line therapies in the future, allowing patients at risk of refractory OAB to significantly benefit.

## Figures and Tables

**Table 1:** Summary of the scientific evidence regarding physical-based agents for OAB. Each device has pros and cons, with specific symptom reduction after using it.

No.	Device	Advantages	Consideration	Long-Term Efficacy	Symptom Reduction
1	P-SNS	- Superior to anticholinergic [18] - Complete continence in some patients [18]	- Needs surgery - Relatively expensive	3–5 years [20,21]	Urgency, frequency, incontinence episodes [19]
2	T-SNS	- Superior to anticholinergic [36,37] - Can be done at home [36]	Needs at least 20 sessions [31] or daily up to 6 months at home [36]	2 years [31]	Nocturia, urgency, incontinence episodes [38]
3	P-TNS	Superior to solifenacin alone; however, the combination of both is more beneficial [46]	- Minimally invasive - Need 12 sessions [46]	3 years [48]	Day and night time frequency, urgency and urge incontinence [46]
4	T-TNS	- Non-invasive - Can be done at home [53]	- Needs 12 sessions [43,53] - May not suitable for patients with poor cognitive capacity [54]	1 years [51]	Nocturia, frequency, incontinence episodes [43]
5	P-MS	- Completely non-invasive - OAB symptoms disappeared in some patients [66] - Patients do not need to be undressed [60]	- Hospital will need a new magnetic chair - Needs 16–18 sessions [65,67]	6 months [67]	Urgency, frequency, urgency incontinence [67]
6	S-MS	- Might be better than T-SNS [72] - General coil of a magnetic stimulation machine can be used	Needs 20 sessions [72]	No data	Urgency, frequency, bladder capacity [71]
7	Laser	- Comparable efficacy to anticholinergic and β3-adrenoceptor agonists [77] - Better than surgery procedures [78] - Only needs 2–3 sessions	Intravaginal procedure [77,78]	<12 months [76]	Frequency, nocturia, urgency, urge incontinence episodes [77,78]
8	Li-ESW	- Non-invasive - ESW machine is usually available in most hospitals	Needs 8 sessions [80,81]	No data	Daytime frequency, urgency, decreased post-voided residual urine, increased bladder capacity [80,81]
